# Outcomes of a Cluster Randomized Controlled Trial of the SoMe Social Media Literacy Program for Improving Body Image-Related Outcomes in Adolescent Boys and Girls

**DOI:** 10.3390/nu13113825

**Published:** 2021-10-27

**Authors:** Chloe S. Gordon, Hannah K. Jarman, Rachel F. Rodgers, Siân A. McLean, Amy Slater, Matthew Fuller-Tyszkiewicz, Susan J. Paxton

**Affiliations:** 1 School of Psychology and Public Health, La Trobe University, Melbourne, VIC 3086, Australia; chloe.gordon@acu.edu.au (C.S.G.); s.mclean@latrobe.edu.au (S.A.M.); susan.paxton@latrobe.edu.au (S.J.P.); 2 ACU Engagement, Australian Catholic University, Melbourne, VIC 3002, Australia; 3 APPEAR, Department of Applied Psychology, Northeastern University, Boston, MA 02115, USA; r.rodgers@northeastern.edu; 4 Department of Psychiatric Emergency & Acute Care, Lapeyronie Hospital, CHRU Montpellier, 34090 Montpellier, France; 5 Centre for Appearance Research, University of West of England, Bristol BS16 1QY, UK; amy.slater@uwe.ac.uk; 6 School of Psychology, Deakin University, Geelong, VIC 3220, Australia; matthew.fuller-tyszkiewicz@deakin.edu.au; 7 Centre for Social and Early Emotional Development, School of Psychology, Deakin University, Melbourne, VIC 3125, Australia

**Keywords:** body image, dietary restraint, wellbeing, social media, RCT, adolescents, eating disorders, school-based prevention

## Abstract

Although the negative effect of social media use among youth on body image and eating concerns has been established, few classroom-based resources that can decrease these effects through targeting social media literacy skills have been developed. This study aimed to test the efficacy of SoMe, a social media literacy body image, dieting, and wellbeing program for adolescents, through a cluster randomized controlled trial. Participants (*n* = 892; M_age_ = 12.77, *SD* = 0.74; range 11–15; 49.5% male) were randomized by school (*n =* 8) to receive either weekly SoMe (*n* = 483) or control sessions (lessons as usual; *n =* 409) over 4 weeks in their classroom. Participants completed surveys at four timepoints (baseline, 1-week post-intervention, and 6- and 12-month follow-up) assessing body dissatisfaction, dietary restraint, strategies to increase muscles (primary outcomes), self-esteem and depressive symptoms (secondary outcomes), and internalization of appearance ideals and appearance comparison (exploratory outcomes). Modest positive intervention effects were found in dietary restraint and depressive symptoms at 6-month follow-up in girls but few positive effects emerged for boys. The findings provide only preliminary support for a social media literacy intervention, but suggest the usefulness of both identifying those who benefit most from a universally delivered intervention and the need to refine the intervention to maximize intervention effects.

## 1. Introduction

Eating disorders are serious mental illnesses that have long-lasting psychological, physical, and economic implications [1,2]. Concerningly, eating disorders are becoming increasingly prevalent [3]. Among the most well-established risk factors for eating disorder onset are body dissatisfaction and dieting behaviors [4]. High levels of body dissatisfaction are reported by adolescents, with one study identifying 42.8% and 14.5% of adolescent girls and boys, respectively, are “extremely” or “very” concerned about their body image [5]. In early adolescents, 19.6% and 6.8% of girls and boys, respectively, reported clinically significant levels of body dissatisfaction [6]. Dieting behaviors are also commonly reported. One study found a very high proportion of adolescents reported having ever engaged in a muscularity-orientated behavior (e.g., lift weights, drink protein shakes) and a little under half in restrictive and binging behaviors [7].

High rates of body dissatisfaction among adolescents are concerning not only due to their potential to predict eating disorder onset but also to predict an array of adverse outcomes, including poor quality of life, psychological distress [8], low self-esteem and depressive symptoms [9,10,11], use of unhealthy body change behaviors [12,13], and health risk behaviors [14,15]. In view of these negative health outcomes, the need for prevention of body dissatisfaction and dieting to interrupt the pathway to further disorders has been emphasized [16,17,18]. As a result, the identification of effective prevention strategies to reduce body dissatisfaction and related body change activities in adolescents is recognized as a vital area of research [16,17].

### 1.1. The Social Media Environment

According to sociocultural theory, three key sociocultural influences impact the development and maintenance of body dissatisfaction: media, parents, and peers [19], with media being identified as one of the most pervasive and salient [20]. With advancements in technology over the last two decades, time spent on digital media (e.g., social media) has now surpassed that of traditional media among adolescents [21]. Social media refers to online platforms that allow the user to create their own profiles and to interact, share, and view content online with others [22]. Social media is especially popular among adolescents, with 85% of adolescents reportedly having a social media account [23]. Adolescents report spending approximately 3 h per day on social media, with scholars labeling social media as a “pervasive and salient developmental context in the daily lives of early adolescents” [24].

Social media provides a unique peer and media environment that enhances pressures to conform to appearance ideals, thereby increasing body dissatisfaction. Much of the content on social media is appearance-focused, typically presenting and reinforcing appearance-ideals [25]. The asynchronicity of social media allows time for careful curating and editing of images before posting online, facilitating a focus on appearance and self-presentation [26]. Social media is also an interactive environment, which allows users to provide and receive feedback. Adolescents report seeking appearance-related comments as a means of validation [27], yet receiving appearance-related commentary on social media has been found to be related to negative body image and restrained eating [28]. Furthermore, social media facilitates widespread opportunities for social comparison, and, given the salience of appearance-related idealized content online, these comparisons typically result in negative appearance self-evaluations [29]. Together, these factors contribute to the influence of social media on body image.

Reviews of cross-sectional research have consistently found higher social media use to be related to higher body dissatisfaction [30,31,32]. In addition, longitudinal evidence suggests a direct effect from social media use to body dissatisfaction [33] as well as the existence of indirect effects through appearance comparisons [34,35]. Experimental research consistently finds that exposure to idealized social media images, as well as the creation of self-images, results in increased body dissatisfaction [36,37,38]. Thus, a robust body of evidence has supported the pathways outlined by sociocultural theories accounting for the detrimental effects of social media use on body image and the role of idealized self-images.

### 1.2. Body Image Interventions

Given the negative impact of idealized social media images on adolescent body image, it is essential to develop and evaluate interventions to counteract this influence. Schools offer appropriate settings for such efforts, with opportunities for high reach of the target population at low cost [39]. A review of school-based body image interventions found that programs were particularly effective among young adolescents (12–13 years) [40]. While girls report higher social media use (i.e., Instagram) than boys, both boys and girls experience appearance pressures on social media and concerns regarding negative appearance evaluation by others [41]. As the relationships between social media use and body satisfaction appear somewhat consistent among adolescent boys and girls [31,42] and there are practical advantages of co-educational delivery, co-educational schools appear to be appropriate settings for interventions.

While limited work has focused on interventions to address the social media environment, a number of interventions exist that have successfully targeted traditional media and peer influences and, therefore, lay useful groundwork for interventions targeting social media effects. Indeed, effective school-based body image interventions typically include health literacy components, namely media literacy [40]. Health literacy refers to skills and capabilities that facilitate understanding and application of health information to improve health and wellbeing [43]. Within this vein, media literacy refers to having skills to evaluate and critically analyze media content. In theory, a more critical appraisal of unrealistic and idealized images reduces their credibility and relevance to the individual, limiting the negative impact of viewing the images [44]. Media literacy interventions have shown effectiveness in improving a range of body image-related outcomes, including the internalization of appearance ideals and weight and shape concerns [45,46,47,48]. Interventions that contain some elements of media literacy have also shown promise in improving body image and reducing internalization of appearance ideals among adolescents [49,50,51,52,53].

### 1.3. Social Media Literacy

Despite their potential, media literacy programs to improve body image are typically limited in their focus to traditional media [47,48,51], which is currently declining in use among this age group [21]. In response to this, an emerging area of research is the examination of the protective role of social media literacy, which refers to skills of critical evaluation of the motives of users’ social media activities. These include that of commercial interests, peers, and celebrities, and presentation practices to present oneself favorably such as picture selection and editing [53]. In support of its protective role in relation to the impact of social media on body image, experimental research has found that commercial social media literacy mitigated the negative effects of exposure to appearance-focused social media content among young adult women, although these findings were not replicated among men [54].

Social media literacy interventions focus on developing social media literacy skills and teach strategies to challenge personal reactions to exposure to social media, including internalization of appearance ideals and social comparison [53]. To date, only a small number of interventions with a primary focus on building social media literacy to reduce body dissatisfaction and disordered eating among adolescents have been evaluated [53,55]. The first, BOOST, was a three-session intervention for Australian adolescent girls aimed at teaching skills to challenge advertising and images presented by social media [53]. In this small, non-randomized study, compared to the control group, those who received the intervention showed improvements in body esteem, dietary restraint, and media literacy from baseline to post-intervention. More recently, Digital Bodies, a single-session body image intervention, which aims to target appearance pressures within the social media environment, has also shown promising findings in the United Kingdom [55]. Adolescents in the intervention group reported improvements in body satisfaction at post-intervention and 8-week follow-up relative to controls. Furthermore, in girls, a reduction in thin-ideal internalization was found at post-intervention in the intervention compared to control group. With preliminary support, it appears that social media literacy interventions may reduce the negative impact of social media use on body image-related outcomes among adolescents. However, these studies were limited by their brief (or lack of) follow-up assessments, and only one [55] included adolescent boys. Consequently, further research is needed to examine the efficacy of a social media literacy intervention among adolescent boys as well as girls within a co-educational setting over an extended period of time in a randomized controlled trial (RCT) design.

### 1.4. The Present Study

Consistent with our protocol [56], the aim of the current research was to evaluate the efficacy of SoMe in improving adolescents’ body image-related outcomes and wellbeing. We hypothesized that participants receiving SoMe would report decreased body dissatisfaction, dietary restraint, and strategies to increase muscles (primary outcome variables), and elevated self-esteem and reduced depressive symptoms (secondary outcomes) one-week post-intervention and from baseline to 6- and 12-months, compared to the no-intervention control group. In addition, change within the intervention group was hypothesized to occur through reduced internalization of appearance ideals and appearance comparison (exploratory outcomes).

## 2. Materials and Methods

### 2.1. Study Design

A two-arm cluster RCT design was used to investigate how social media literacy mitigates the negative impact of social media use in adolescents. Eight schools in Victoria, Australia, of which five were public and three independent, participated in this research. Participants completed a structured self-report questionnaire, which was administered in classroom sessions on four occasions: at baseline (pre-intervention); one-week post-intervention (5-weeks post-baseline); and 6- and 12-months post-baseline. This trial was registered with the Australian New Zealand Clinical Trials Registry (ACTRN12617000137392; www.anzctr.org.au; accessed on 25 January 2017). Further detail on the study rationale and design can be found in the protocol paper [56]. A priori power analysis revealed that a proposed sample size of 350 boys and 350 girls, allowing for 30% attrition, would have sufficient power to detect small to medium effects (*d* = 0.30) between groups.

### 2.2. Participants

Participants were Grade 7 and Grade 8 students (11–15 years of age) attending school in November 2017–August 2019. If their grade was selected to receive SoMe, all students in the grade received the 4-lesson program as part of their school curriculum. However, only students whose parents provided consent were eligible to participate in the research. Most schools required written active parental consent (*n* = 6), while two schools used informed, opt-out parental consent whereby parents could actively choose to withdraw their child from the research. Both consent options were in accordance with ethics approval. A total of 892 participants completed at least one survey over the four timepoints (*n* = 775 at baseline, *n* = 740 at post-intervention, *n* = 700 at 6-month follow-up, and *n* = 685 at 12-month follow-up). At baseline, the average age of participants was 12.77 years (*SD* = 0.74; range 11–15), with 49.5% identifying as male, 49.0% as female, and 1.5% reporting ‘other’/’I would prefer not to respond’. The majority of participants (84.21%) were born in Australia or New Zealand, 8.00% in East and Southeast Asia, and 3.61% in European countries. A small number of participants were born in other countries (4.18%). Participants also reported that their parents were primarily born in Australia or New Zealand (mothers 63.32%; fathers 62.62%), as well as East and Southeast Asia (mothers 18.89%; fathers 17.66%), European countries (mothers 9.65%; fathers 10.48%), or elsewhere (mothers 8.15%; fathers 9.24%). Participants primarily lived in areas of low-relative disadvantage (81.01%), with lower rates in mid- (8.31%) and high-relative disadvantage (10.68%) [57]. Grade levels within schools were randomly assigned to receive either SoMe (*n* = 483) or the control condition, health lessons as usual (*n* = 409).

### 2.3. Intervention

SoMe (a social media literacy body image, dieting, and wellbeing program for early high school students) consisted of four 50-min lessons. It was underpinned by an aetiological perspective, which proposes that a reduction in causal risk factors or increase in protective factors for a problem will disrupt the developmental sequence, resulting in a reduced likelihood that the problem will develop [58]. The tripartite influence model and supporting empirical research indicates a negative impact of exposure to appearance-focused media on body image [19,32]. Based on this, SoMe endeavored to provide early adolescents with critical social media literacy skills to counteract detrimental effects of social media exposure.

Grounded in media literacy principles [59] and constructivist learning theory [60], the lessons were experiential and interactive, and aimed to empower adolescents with skills to critique social media postings, including advertising, celebrity postings, and friends’ personal pages. Students considered realism in social media, developed strategies to respond to negative feedback, explored how social media can be used to bring about positive social change, and importantly, reflected on and practiced how to present their ‘real’ selves on social media with less emphasis placed on appearance. The intervention was delivered by trained facilitators who each received two half-day training workshops run by one of the authors who developed the intervention. In addition, each facilitator observed a session of the intervention being delivered by one of the authors. Finally, facilitator manuals were used to ensure consistency in lesson delivery across classes. For further detail on the program’s theoretical basis, development, and structure and content, including a summary of the intervention’s objectives and learning experiences, see [56].

### 2.4. Measures

Demographic data were obtained, including gender, age, country of birth, language spoken at home, and residential postcode. Primary, secondary, and exploratory outcomes and their internal reliability are outlined in Table 1. These measures have previously shown good validity and reliability in adolescent samples, details of which are presented elsewhere [56].

### 2.5. Procedure

**Figure 1:**
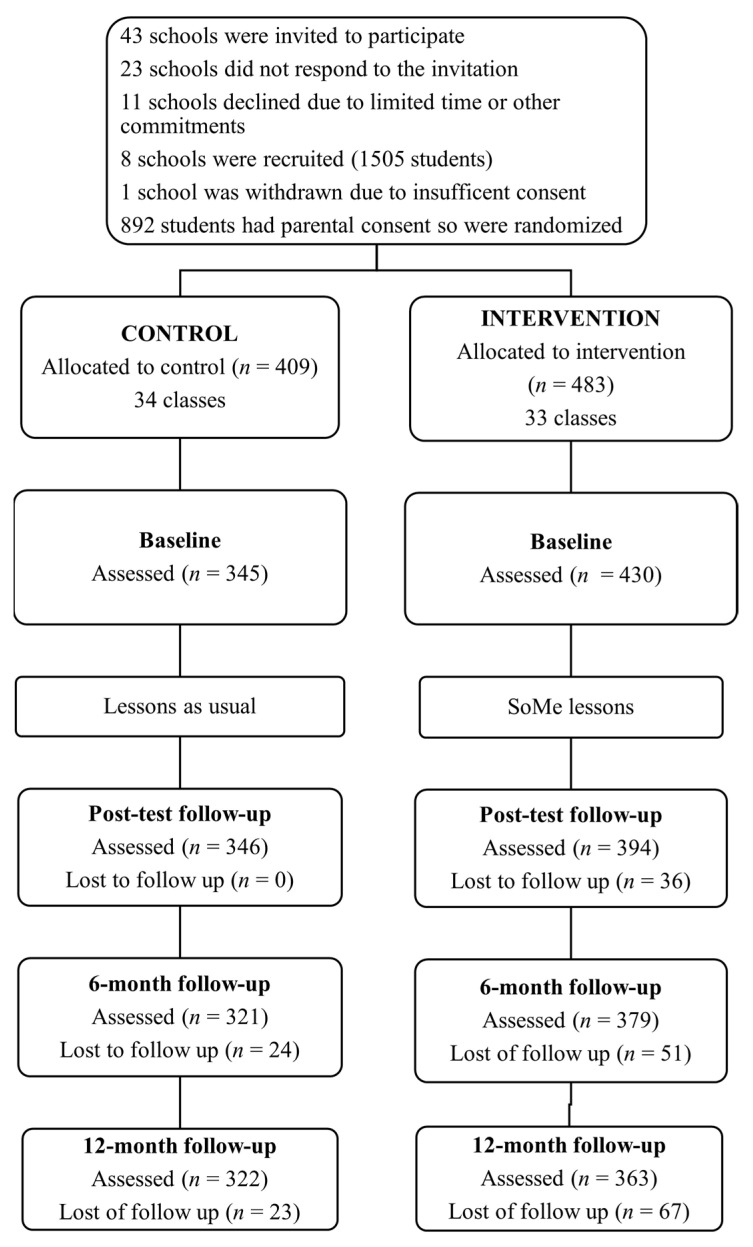
Summary of participant flow.

The research protocol was approved by the La Trobe University Human Ethics Committee (HEC17-020) and the Victorian Department of Education and Training (2017_003388). Blocked randomization was conducted using an online program. Grade 7 and 8 cohorts within each participating school were randomly assigned to one of two conditions: intervention or control. Participants in the intervention condition received four lessons delivered during class time over 4 weeks while the control condition received lessons as usual. All participants were invited to complete questionnaires at baseline (pre-intervention); one-week post-intervention (5-week post-baseline); and 6- and 12-month post-baseline. Data collections were completed during class time, facilitated by a member of the research team alongside supervision from the class teacher. Figure 1 shows participant flow.

### 2.6. Statistical Analysis

Data screening and preliminary analyses were conducted in SPSS version 27. Outcome variables were screened for missing values, outliers, and normality. Scale scores were marked as missing for adolescents who responded to less than 70% of items on a scale.

The main analyses were undertaken using Stata version 16 and conducted on an intention-to-treat basis, with participant data included as per initial intervention group allocation. Linear mixed models were used for all outcome measures, with repeated measurements clustered within individuals. While the study protocol had indicated that internalization of appearance ideals and appearance comparison would mediate changes in outcome variables in the intervention group [56], few significant intervention effects were found. As the intervention aimed to reduce these variables originally indicated as mediating variables, the authors decided to run exploratory analyses with these as outcome variables, as per the main analyses. The final outcome measures were body dissatisfaction, dietary restraint, and strategies to increase muscles (primary outcome variables), self-esteem and depressive symptoms (secondary outcomes), and internalization of appearance ideals and appearance comparison (exploratory outcomes).

Time effects were coded as comparisons between the baseline measurements and all subsequent measurements (i.e., baseline vs. post-intervention, baseline vs. 6-months, baseline vs. 12-months) and were treated as random effects to facilitate evaluation of moderation effect of time by group. An unstructured covariance matrix was applied to most accurately model covariances among these random effects. Preliminary testing was conducted to evaluate clustering effects of school, but all random effects for school were close to zero, and in some instances failed to converge. Accordingly, no adjustment for school was applied in the final analyses.

Analyses were run on the full sample, followed by evaluation of individuals who completed the study (“completers”, operationalized as data provided at all waves) and subgroup analyses for boys and girls. Models are reported in adjusted form, using demographic variables as covariates if identified as differing at baseline between groups. As the research hypotheses address different segments of the overall sample (full sample; completers; boys; girls), models may differ in the covariates included-adjustment variables are identified in relevant tables of results. Effect sizes are reported as standardized mean differences, with values of 0.20 considered small, 0.50 moderate, and 0.80 and above considered large [71]. Across all variables, the amount of missing data ranged from 0 to 26%. Therefore, missing data were handled using multiple imputations (MI) with 50 imputations in all models.

## 3. Results

### 3.1. Preliminary Analyses

Table 2 presents group differences in baseline characteristics. Participants in the control group were significantly younger, less likely to come from public schools, and less likely to report English as their primary language spoken at home. No differences between groups were found for BMI, gender distribution, Indigenous status, or whether participants completed all surveys.

### 3.2. Intervention Efficacy

#### 3.2.1. Full Sample

Table 3 shows intervention effects for the full sample, adjusting for age, language, and school type. The only outcome measure to show different rates of change across groups was drive for increased muscularity (*d* = 0.19). Although there was a decline in drive for muscularity from baseline to 12-month follow-up for the sample overall (*b* = −1.31, *p* < 0.001), unexpectedly, this decline was weaker for individuals in the intervention arm of the study.

#### 3.2.2. Completers

As shown in Supplementary Table S1, a similar pattern emerged when the sample was limited to study completers. Contrary to expectations, controlling for school type, the decline in drive for muscularity from baseline to the 12-month follow-up was weaker for individuals in the intervention group relative to the control condition (*d* = 0.27).

#### 3.2.3. Girls

Two significant group-by-time effects emerged when models were run separately for girls—see Table 4. First, whereas the control group slightly increased in dietary restraint from baseline to the 6-month follow-up, the intervention group exhibited reduced symptoms (*d* = 0.24). Second, increases in depressive symptoms experienced for the sample overall from baseline to the 6-month follow-up were weaker for girls in the intervention arm than the control arm (*d* = 0.22).

#### 3.2.4. Boys

Three significant group-by-time effects were observed for boys—see Table 5. First, whereas dietary restraint behaviors reduced from baseline to the 12-month follow-up for both groups, unexpectedly, this effect was weaker for the intervention arm (*d* = 0.23). Second, contrary to expectations, the intervention group exhibited an increase in drive for muscularity from baseline to the 12-month follow-up, while the control group showed a decline in this behavior (*d* = 0.29). Third, as predicted, the intervention group showed an increase in self-esteem from baseline to the 6-month follow-up, whereas self-esteem scores declined for the control group (*d* = 0.29).

## 4. Discussion

The present study evaluated the efficacy of a social media literacy intervention (SoMe) in bringing about positive change in adolescents’ body image, body change strategies, and wellbeing. Contrary to our hypotheses, for the total sample, the intervention was not associated with improvements in body dissatisfaction, dietary restraint, self-esteem, depressive symptoms, internalization, or comparisons relative to the control group. The only difference to emerge in the full and completers sample was for drive for muscularity, whereby weaker declines were found in the intervention group relative to the control group. While the intervention showed benefits for girls in terms of dietary restraint and depressive symptoms at 6-month follow-up relative to controls, it appeared less effective among boys.

The findings indicate only minor intervention effects within the full group and among completers. These findings are somewhat consistent with those of previous research, which found improvements across a small number of outcomes [53,55]. These small effects may be attributable to a number of factors. First, although the intervention has theoretical grounding, was developed by a team with recognized expertise in the field, and included input from teachers, participants may have had difficulty in translating the skills imparted in the intervention. It is possible that the participants (aged 12–13 years) may have struggled with critical thinking skills and their ability to apply the intervention concepts to real life. If so, additional work may be needed to support adolescents to build and apply these skills over time. Alternatively, targeting interventions among older adolescents may be appropriate as they may be more capable of critical thinking skills. In regards to intervention delivery, all facilitators received training and used an intervention manual to present the lessons. Despite this, it is possible that the content and messages within the intervention were not delivered effectively. Perhaps external facilitators are not as effective at delivering this type of content to adolescents, in which case research should explore the effectiveness of delivery by class teachers. The established relationship between teachers and students may make adolescents more receptive to content and the regular classroom teachers will be familiar with the specific needs of students and, therefore, more likely to deliver the content in a way that resonates with students.

Participant engagement, motivation, and initiative to change may have been low in the present sample, resulting in fewer intervention effects. Given the pervasiveness of social media and how engrained it is in the lives of adolescents, it is likely that adolescents may demonstrate some resistance, or not be motivated, to changing their social media habits. Relatedly, scholars have begun to question whether traditional approaches within media literacy interventions can mitigate against social media effects on body dissatisfaction [72]. Shuilleabhain and colleagues suggest, for example, that while young people may demonstrate an awareness that social media images are edited, this does not necessarily mean that they are able to or have a want to disengage from the desire to achieve such appearances. This suggestion is supported by findings from experimental work showing that despite being able to recognize social media images as edited, adolescent girls rate manipulated images as highly attractive [36]. Shuilleabhain and colleagues argue that media literacy interventions should move beyond rationalistic approaches, where the onus is on the individual to separate social media from the self, to embodied, affective processes, which consider the relational experience of social media and its interconnectedness with the self among young people. Additional theoretical and empirical research is necessary to explore this concept further.

Furthermore, a number of considerations related to the nature and power of social media platforms themselves should be considered and contrasted with the intervention dose. As articulated above, social media platforms can be viewed as combining peer and traditional media influences. They are also extremely attractive to youth, who are motivated to engage with them for a host of reasons, including communicating with their friends, but also entertainment, social capital, and identity development [73]. These underlying motivations constitute powerful influences on the ways in which youth engage with social media. In addition, social media platforms are profit-driven industries, so much effort is invested into designing experiences that are appealing and promote the consumption of content. Relatedly, social media has been described as difficult to disengage from, reflecting the potency of the algorithms and the functionalities that engage users [74]. Finally, while this intervention was delivered at the classroom level, participants likely interact with other social groups on social media and would therefore likely continue to experience pressure to engage in their habitual social media practices. Faced with these powerful combinations of factors promoting engagement with social media in ways that are at odds with the tenets of social media literacy, it may be that a larger intervention dose is necessary to achieve the intended effects.

When the intervention effects were examined among girls, participants in the intervention group reported a reduction in dietary restraint at 6-month follow-up, whereas the control condition indicated increased dietary restraint. Further, increases in depressive symptoms among girls at 6-month follow-up were less pronounced for participants in the intervention relative to control condition. These findings suggest that the intervention is somewhat beneficial for girls. A reduction in dietary restraint is especially promising given that dieting behaviors are one of the most established risk factors for eating disorder onset [4]. However, both intervention effects were only evident from baseline to 6-month follow-up indicating that effects were not maintained at 12-month follow-up. One option to maintain intervention effects over time could be to provide booster sessions. In addition, although the present study used trained facilitators, interventions that train teachers to deliver this content may produce longer lasting effects given the continued interaction between teachers and students.

For boys, a number of effects were found, with mixed findings. First, although the sample reported overall reductions in dietary restraint from baseline to 12-month follow-up, declines were weaker in the intervention group relative to control. Further, the intervention group demonstrated increased self-esteem and drive for muscularity from baseline to 6-months and 12-month follow-up, respectively, while the control group declined. Although increased self-esteem is a promising outcome of the intervention, increased drive for muscularity is not. It is possible that presenting examples of muscular appearances within the intervention may have inadvertently promoted upward comparisons with these images. However, given that such content is widespread in society, including on social media [75], it is unlikely that this would have had a considerable effect. The present study is the first that the authors are aware of that examined the efficacy of a school-based social media literacy intervention specific to eating behaviors among boys, so additional research is required.

The intervention appears to be slightly more efficacious among girls compared to boys. Social media literacy has been found to be protective against body dissatisfaction among young women but not men [54], so it is possible that interventions designed to enhance social media literacy will be less effective among boys. In line with the present study, eating disorder prevention interventions are more beneficial for girls than boys [18]. Girls engage in more appearance-focused social media use behaviors [76] and report poorer body image outcomes [6] than boys, so the present intervention may have been of greater relevance to girls suggesting the need for more research to explore options to enhance the effectiveness of social media literacy interventions to improve body image-related outcomes among boys.

The present study has a number of strengths, including the randomized controlled trial design, a diverse sample of adolescents from a range of school demographics (including both state and private secondary schools), and a longer follow-up period than previous social media literacy interventions. However, there were some limitations to acknowledge. First, sample attrition was high at approximately 30% at 12-month follow-up. However, the analyses on the full sample and completers showed the same pattern of results. Second, although the present study extended previous research by using longer follow-up periods (i.e., 6- and 12-months), future research would benefit from follow-up beyond 1 year.

The present study found modest support for SoMe in reducing dietary restraint and improving wellbeing in early adolescents and provides some evidence of the usefulness of a universal in-class intervention. Although the effects were small, this is not unexpected in the context of a universal intervention [40]. Given the far-reaching negative impacts of social media use among adolescents, even small effects may have scalable benefits. Furthermore, although clinical eating disorders affect all genders, they continue to have a higher prevalence among girls [6], so the fact that SoMe was successful in reducing dieting among girls, albeit with a small effect, is therefore noteworthy.

These findings suggest a number of important directions for future research. The first is the extension of the work and efforts to identity for whom the intervention may be most useful by exploring individual moderating factors. Relatedly, decision tree analyses could be explored to identify subgroups within the sample who benefitted substantially from the intervention. Findings from such work will further inform the cost-benefit ratio of a universal prevention approach to the reduction of body image and eating concerns through social media literacy. In addition, future research should aim to identify ways to increase the intervention dose and potentialize intervention effects. As mentioned above, booster sessions, or further integration of the content into existing curriculums might achieve this. Further, it could be fruitful to pair educational-style interventions with technology-based interruptions such that youth are further nudged when using social media [77]. Finally, considering how to incorporate perceptions of peer norms into the intervention to decrease potential concerns regarding the negative impacts on social relationships of implementing the strategies included in the intervention may be useful [78].

As parents and educators continue to call for social media literacy resources to address its impact on body image, dieting, and wellbeing (and indeed introduce programs that have not been evaluated and may have unidentified risks), it is essential to find evidence-based strategies that are effective. Thus, more work is needed to address this. Further, given rates of social media use among adolescents have increased during the COVID-19 pandemic [79], there is urgency in conducting this research and providing evidence-based interventions.

## 5. Conclusions

Demand for social media body image interventions for adolescents is high. The present study tested a new school-based social media literacy intervention, aiming to fill the gap in available universal resources for youth. Findings were overall modest in terms of intervention effects yet notable given the brief nature of the intervention and its universal nature. Specifically, findings among girls indicated that the intervention was valuable in addressing dietary restraint and depressive symptoms at the 6-month follow-up. Fewer positive effects emerged among boys. Together, these findings provide preliminary support for school-based social media literacy interventions, particularly among adolescent girls.

## Figures and Tables

**Table 1:** Overview of the instruments used to evaluate the efficacy of the SoMe intervention.

Outcome Measures		Construct	Cronbach Alpha
Primary outcome variables	12-item Weight and Shape Concern subscales of the Eating Disorder Examination Questionnaire (EDE-Q) [61] e.g., “Have you had a strong desire to lose weight?”	Body dissatisfaction	0.96
	Three 100-point visual analogue scales (VAS) [62] e.g., “I feel satisfied with my body shape”	State body dissatisfaction	-
	10-item Restraint subscale from the Dutch Eating Behaviour Questionnaire (DEBQ) [63] e.g., “Do you deliberately eat foods that are slimming?”	Dietary restraint	0.94
	5-item Strategies to Increase Muscle Size subscale from the Body Change Inventory (BCI) [64] e.g., “How often do you think about changing your level of exercise to increase the size of your muscles?”	Strategies to increase muscles	0.92
Secondary outcome variables	9-items from the Centre for Epidemiological Studies Depression Scale Revised (CESDR-10) [65] e.g., “I had trouble keeping my mind on what I was doing”	Depressive symptoms	0.90
	Single-item Self-Esteem Scale [66] 3-items from the Rosenberg Self-Esteem Scale [67] e.g., “I feel I have a number of good qualities”	Self-esteem	0.71
Exploratory outcome variables	5-items (adapted) from The Sociocultural Attitudes Towards Appearance Scale-3 (SATAQ-3) [68] e.g., “I would like my body to look like people on social media”	Internalization of social media appearance ideals	0.81
	5-item Internalization-Muscular/Athletic Subscale of the Sociocultural Attitudes Towards Appearance Scale-4 (SATAQ-4) [69] e.g., “It is important for me to look athletic”	Internalization of muscular/athletic ideals	0.93
	5-items from the Upward Physical Appearance Comparison Scale (UPACS) [70] e.g., “I tend to compare myself to people I think look better than me”	Upward appearance comparison	0.97

Internal reliability of scale scores is represented by baseline measurement.

**Table 2:** Demographic differences at baseline between intervention and control groups.

	Intervention		Control		Difference	
	*M*	*SD*	*M*	*SD*	*t*	*p*-Value
Age	12.768	0.571	12.648	0.853	−2.08	0.038
BMI	18.997	2.617	19.210	2.681	0.44	0.662
	*n*	%	*n*	%	*x* ^2^	*p*-value
Completers:					0.610	0.435
Yes	259	53.63	230	56.23		
No	224	46.38	179	43.77		
School type:					25.65	<0.001
Independent	145	55.98	160	69.57		
Public	114	44.02	70	30.43		
Gender:					1.29	0.732
Male	129	49.81	108	46.96		
Female	128	49.42	117	50.87		
Other	2	0.77	3	1.30		
Prefer not to respond	0	0	2	0.87		
Primary language spoken at home:					15.59	0.001
English	210	81.08	197	85.65		
Other	37	14.29	24	10.43		
English and other	9	3.47	7	3.04		
Not specified	3	1.16	2	0.87		
Indigenous background:					3.42	0.332
Aboriginal	1	0.39	2	0.87		
Torres Strait Islander	2	0.77	1	0.43		
No	256	98.84	227	98.70		

**Table 3:** Mean changes in study variables at post-intervention and 6- and 12-month follow-up for main analyses.

	Study Group				Difference in Change Score		
	Control		Intervention		(Group 1—Group 0)		
Outcome	*n*	M (SD)	*n*	M (SD)	M [95% CI]	Effect Size	*p* (Two-Tailed)
**Primary and secondary outcomes**							
Weight/shape concerns							
Baseline	343	1.68 (1.70)	418	1.59 (1.65)			
Post-intervention	342	1.56 (1.64)	387	1.47 (1.62)	0.00 [−0.16, 0.17]	0.00	0.998
6-month follow-up	316	1.67 (1.67)	373	1.68 (1.70)	0.08 [−0.12, 0.27]	0.047	0.449
12-month follow-up	319	1.85 (1.75)	361	1.76 (1.65)	0.05 [−0.17, 0.26]	0.029	0.656
State body satisfaction							
Baseline	342	64.20 (31.97)	418	64.96 (32.02)			
Post-intervention	342	65.17 (32.53)	387	67.75 (30.69)	1.54 [−1.90, 4.97]	0.048	0.381
6-month follow-up	316	66.16 (31.71)	373	67.10 (30.78)	0.09 [−3.66, 3.83]	0.002	0.963
12-month follow-up	318	62.21 (31.98)	358	65.68 (30.70)	2.03 [−2.27, 6.32]	0.063	0.355
Dietary restraint							
Baseline	341	1.95 (0.97)	424	1.96 (1.00)			
Post-intervention	341	1.81 (0.97)	388	1.82 (0.96)	0.03 [-.008, 0.13]	0.030	0.633
6-month follow-up	316	1.92 (1.04)	374	1.87 (0.97)	−0.06 [−0.20, 0.07]	−0.060	0.334
12-month follow-up	316	1.95 (1.04)	361	1.90 (1.01)	0.03 [−0.11, 0.18]	0.030	0.650
Drive to increase muscularity							
Baseline	341	11.23 (5.33)	423	11.05 (5.54)			
Post-intervention	343	10.38 (5.35)	386	10.26 (5.31)	0.17 [−0.45, 0.78]	0.031	0.596
6-month follow-up	319	10.48 (5.31)	374	10.56 (5.16)	0.45 [−0.32, 1.21]	0.082	0.254
12-month follow-up	319	10.24 (5.22)	360	10.56 (5.01)	1.01 [0.16, 1.87]	0.185	0.020
Self-esteem							
Baseline	340	11.09 (3.06)	415	10.86 (2.80)			
Post-intervention	339	11.31 (3.00)	383	10.81 (3.03)	−0.16 [−0.52, 0.20]	−0.054	0.376
6-month follow-up	319	10.88 (2.99)	370	10.87 (3.11)	0.22 [−0.20, 0.65]	0.075	0.305
12-month follow-up	314	10.69 (3.24)	358	10.62 (3.07)	0.18 [−0.30, 0.65]	0.061	0.464
Depressive symptoms							
Baseline	337	16.28 (7.51)	418	15.91 (7.52)			
Post-intervention	341	16.06 (7.50)	388	16.20 (8.05)	0.30 [−0.61, 1.22]	0.039	0.515
6-month follow-up	319	17.35 (8.60)	372	16.42 (8.10)	−0.73 [−1.78, 0.33]	−0.097	0.177
12-month follow-up	316	17.79 (8.59)	357	18.13 (8.38)	0.90 [−0.22, 2.02]	0.119	0.115
**Exploratory outcomes**							
Thin ideal internalization							
Baseline	342	11.61 (5.19)	424	12.00 (5.13)			
Post-intervention	340	11.81 (5.02)	388	11.97 (4.70)	−0.11 [−0.73, 0.50]	−0.021	0.722
6-month follow-up	315	12.44 (5.10)	374	11.91 (4.99)	−0.67 [−1.38, 0.04]	−0.130	0.063
12-month follow-up	317	12.58 (5.19)	358	12.58 (4.88)	−0.10 [−0.78, 0.58]	−0.019	0.771
Muscular internalization							
Baseline	341	11.81 (5.66)	427	11.42 (5.31)			
Post-intervention	343	11.28 (5.76)	389	10.97 (5.32)	−0.14 [−0.81, 0.52]	−0.026	0.671
6-month follow-up	318	11.65 (5.67)	374	11.55 (5.60)	0.25 [−0.52, 1.02]	0.046	0.52
12-month follow-up	318	11.58 (5.73)	359	11.84 (5.56)	0.71 [−0.14, 1.56]	0.130	0.100
Upwards appearance comparison							
Baseline	341	11.81 (6.66)	422	11.24 (6.35)			
Post-intervention	339	11.36 (6.61)	389	11.27 (6.15)	0.53 [−0.18, 1.24]	0.082	0.142
6-month follow-up	318	12.29 (6.88)	372	11.64 (6.43)	−0.04 [−0.89, 0.81]	−0.006	0.925
12-month follow-up	317	12.73 (6.83)	359	12.15 (6.60)	0.34 [−0.59, 1.28]	0.052	0.468

Effects are based on multiple imputations *(n =* 50 imputations). Differences compare post-intervention to all other timepoints, adjusted for age, language, and school type.

**Table 4:** Mean changes in study variables at post-intervention and 6- and 12-month follow-up for girls only.

	Study Group				Difference in Change Score		
	Control		Intervention		(Group 1—Group 0)		
Outcome	*n*	M (SD)	*n*	M (SD)	M [95% CI]	Effect Size	*p* (Two-Tailed)
**Primary and secondary outcomes**							
Weight/shape concerns							
Baseline	174	2.12 (1.76)	196	2.05 (1.81)			
Post-intervention	172	2.06 (1.77)	182	1.77 (1.68)	−0.20 [−0.43, 0.02]	−0.112	0.074
6-month follow-up	157	2.23 (1.79)	191	2.15 (1.84)	−0.02 [−0.29, 0.25]	−0.011	0.864
12-month follow-up	162	2.56 (1.79)	165	2.41 (1.70)	−0.08 [−0.41, 0.25]	−0.045	0.637
State body satisfaction							
Baseline	173	57.42 (31.74)	195	60.47 (32.50)			
Post-intervention	172	58.15 (33.14)	181	64.36 (30.94)	2.74 [−1.73, 7.21]	0.085	0.229
6-month follow-up	158	57.44 (31.54)	192	60.78 (32.48)	0.53 [−4.95, 6.00]	0.016	0.850
12-month follow-up	162	51.21 (30.38)	164	57.73 (32.10)	2.89 [−3.32, 9.10]	0.090	0.361
Dietary restraint							
Baseline	173	2.05 (1.01)	202	2.13 (1.09)			
Post-intervention	172	1.93 (1.01)	183	1.90 (0.98)	−0.11 [−0.25, 0.03]	−0.104	0.111
6-month follow-up	157	2.10 (1.10)	192	1.97 (1.02)	−0.25 [−0.42, −0.07]	−0.237	0.005
12-month follow-up	160	2.26 (1.12)	165	2.18 (1.09)	−0.13 [−0.34, 0.07]	−0.123	0.209
Drive to increase muscularity							
Baseline	173	10.53 (4.77)	200	10.92 (5.67)			
Post-intervention	174	9.84 (4.76)	181	9.65 (5.06)	−0.45 [−1.31, 0.41]	−0.085	0.305
6-month follow-up	160	9.81 (4.62)	192	10.01 (5.06)	−0.13 [−1.04, 0.78]	−0.025	0.781
12-month follow-up	162	9.62 (4.25)	165	9.87 (4.81)	0.22 [−0.86, 1.30]	0.042	0.695
Self-esteem							
Baseline	174	10.57 (3.15)	197	10.66 (2.93)			
Post-intervention	172	10.90 (3.10)	183	10.52 (3.24)	−0.35 [−0.84, 0.14]	−0.115	0.157
6-month follow-up	160	10.69 (2.96)	192	10.55 (3.15)	−0.18 [−0.73, 0.37]	−0.059	0.516
12-month follow-up	161	9.99 (3.16)	164	10.18 (3.07)	0.05 [−0.60, 0.71]	0.016	0.873
Depressive symptoms							
Baseline	172	17.50 (7.85)	199	17.02 (8.32)			
Post-intervention	172	16.91 (7.26)	184	16.95 (8.37)	0.08 [−1.18, 1.33]	0.010	0.905
6-month follow-up	160	19.54 (9.10)	192	17.73 (8.58)	−1.77 [−3.43, −0.10]	−0.218	0.038
12-month follow-up	162	19.85 (8.80)	164	19.79 (8.87)	0.74 [−0.98, 2.47]	0.091	0.398
**Exploratory outcomes**							
Thin ideal internalization							
Baseline	174	12.60 (5.41)	201	12.88 (5.50)			
Post-intervention	172	12.49 (5.37)	183	12.60 (4.82)	−0.16 [−0.98, 0.65]	−0.029	0.694
6-month follow-up	157	13.27 (5.31)	192	12.73 (5.28)	−0.76 [−1.72, 0.20]	−0.139	0.119
12-month follow-up	161	14.12 (5.04)	164	13.60 (4.96)	−0.62 [−1.62, 0.37]	−0.114	0.220
Muscular internalization							
Baseline	173	10.84 (5.26)	203	10.60 (5.00)			
Post-intervention	173	10.53 (5.24)	183	9.94 (4.78)	−0.54 [−1.34, 0.27]	−0.105	0.192
6-month follow-up	159	10.81 (5.02)	192	10.60 (5.20)	−0.07 [−1.08, 0.94]	−0.014	0.896
12-month follow-up	162	11.18 (5.19)	165	10.98 (5.19)	0.05 [−1.11, 1.21]	0.010	0.932
Upwards appearance comparison							
Baseline	173	13.99 (6.76)	202	12.89 (6.72)			
Post-intervention	170	13.08 (6.89)	183	12.42 (6.49)	0.37 [−0.64, 1.38]	0.055	0.470
6-month follow-up	159	14.33 (7.05)	191	13.28 (6.58)	−0.25 [−1.46, 0.95]	−0.037	0.677
12-month follow-up	161	15.44 (6.66)	165	14.32 (6.76)	−0.01 [−1.34, 1.31]	−0.001	0.988

Effects are based on multiple imputations (*n =* 50 imputations). Differences compare post-intervention to all other timepoints, adjusted for age, language, and school type.

**Table 5:** Mean changes in study variables at post-intervention and 6- and 12-month follow-up for boys only.

	Study Group				Difference in Change Score		
	Control		Intervention		(Group 1—Group 0)		
Outcome	*n*	M (SD)	*n*	M (SD)	M (95% CI)	Effect Size	*p* (Two-Tailed)
**Primary and secondary outcomes**							
Weight/shape concerns							
Baseline	163	1.15 (1.41)	217	1.14 (1.35)			
Post-intervention	165	1.03 (1.29)	202	1.20 (1.52)	0.18 [−0.04, 0.41]	0.131	0.116
6-month follow-up	153	1.10 (1.31)	179	1.18 (1.37)	0.14 [−0.10, 0.38]	0.102	0.261
12-month follow-up	150	1.05 (1.28)	193	1.19 (1.38)	0.19 [−0.08, 0.46]	0.138	0.160
State body satisfaction							
Baseline	163	71.44 (30.33)	217	69.34 (30.89)			
Post-intervention	165	72.43 (30.19)	203	70.73 (30.22)	−0.23 [−5.52, 5.05]	−0.008	0.932
6-month follow-up	152	75.26 (29.19)	178	73.92 (27.39)	−0.66 [−6.46, 5.14]	−0.022	0.823
12-month follow-up	149	74.35 (29.03)	191	72.75 (27.44)	−0.31 [−6.56, 5.94]	−0.010	0.922
Dietary restraint							
Baseline	162	1.82 (0.88)	216	1.79 (0.88)			
Post-intervention	164	1.69 (0.89)	202	1.75 (0.92)	0.13 [−0.01, 0.28]	0.148	0.074
6-month follow-up	153	1.73 (0.92)	179	1.75 (0.88)	0.09 [−0.10, 0.28]	0.102	0.338
12-month follow-up	149	1.59 (0.81)	193	1.67 (0.87)	0.20 [0.00, 0.40]	0.227	0.049
Drive to increase muscularity							
Baseline	162	11.90 (5.65)	217	11.11 (5.42)			
Post-intervention	164	10.96 (5.87)	202	10.79 (5.50)	0.72 [−0.19, 1.64]	0.130	0.122
6-month follow-up	153	11.23 (5.89)	179	11.12 (5.17)	0.91 [−0.24, 2.06]	0.165	0.123
12-month follow-up	150	10.93 (6.03)	192	11.08 (5.06)	1.61 [0.41, 2.81]	0.292	0.008
Self-esteem							
Baseline	160	11.74 (2.79)	213	11.06 (2.65)			
Post-intervention	162	11.85 (2.77)	198	11.10 (2.77)	0.06 [−0.47, 0.59]	0.022	0.821
6-month follow-up	153	11.13 (3.01)	175	11.26 (3.02)	0.78 [0.17, 1.39]	0.288	0.012
12-month follow-up	146	11.46 (3.13)	191	11.02 (3.00)	0.38 [−0.27, 1.03]	0.140	0.254
Depressive symptoms							
Baseline	159	14.65 (6.47)	214	14.78 (6.36)			
Post-intervention	164	15.10 (7.64)	202	15.42 (7.50)	0.08 [−1.31, 1.47]	0.012	0.910
6-month follow-up	153	14.86 (7.23)	177	14.95 (7.17)	0.17 [−1.21, 1.56]	0.027	0.807
12-month follow-up	147	15.40 (7.60)	190	16.62 (7.64)	1.31 [-.21, 2.82]	0.204	0.091
**Exploratory outcomes**							
Thin ideal internalization							
Baseline	162	10.50 (4.77)	218	11.14 (4.60)			
Post-intervention	163	11.04 (4.58)	203	11.44 (4.53)	−0.09 [−0.97, 0.79]	−0.019	0.843
6-month follow-up	152	11.65 (4.81)	179	11.07 (4.47)	−0.79 [−1.77, 0.18]	−0.169	0.112
12-month follow-up	149	10.93 (4.84)	191	11.69 (4.58)	0.37 [−0.64, 1.39]	0.079	0.469
Muscular internalization							
Baseline	162	12.69 (5.81)	218	12.11 (5.48)			
Post-intervention	165	12.11 (6.16)	204	11.89 (5.60)	0.21 [−0.79, 1.21]	0.037	0.682
6-month follow-up	153	12.61 (6.19)	179	12.58 (5.79)	0.53 [−0.62, 1.69]	0.094	0.363
12-month follow-up	149	12.00 (6.31)	191	12.60 (5.79)	1.34 [.08, 2.60]	0.238	0.037
Upwards appearance comparison							
Baseline	162	9.27 (5.42)	214	9.60 (5.52)			
Post-intervention	164	9.55 (5.81)	204	10.22 (5.63)	0.56 [−0.46, 1.59]	0.102	0.281
6-month follow-up	153	10.31 (6.02)	178	9.89 (5.71)	−0.24 [−1.31, 0.83]	−0.044	0.664
12-month follow-up	149	9.87 (5.81)	191	10.18 (5.71)	0.37 [−0.80, 1.55]	0.068	0.531

Effects are based on multiple imputations (*n =* 50 imputations). Differences compare post-intervention to all other timepoints, adjusted for age, language, and school type.

## Data Availability

The data presented in this study are available on request from the corresponding author. The data are not publicly available due to ethical restrictions.

## Supplementary Materials

The following are available online at https://www.mdpi.com/2072-6643/13/11/3825/s1; Table S1: Mean change in study variables at post-intervention and 6- and 12-month follow-up adjusted for ‘completers’.
