# Supplementary material for: Outcomes of a Cluster Randomized Controlled Trial of the SoMe Social Media Literacy Program for Improving Body Image-Related Outcomes in Adolescent Boys and Girls
Source: Nutrients. 2021 Oct 27;13(11):3825. doi: 10.3390/nu13113825 (PMC8674763; doi:10.3390/nu13113825)
Supplement: Supplementary file 1 [file nutrients-13-03825-s001.zip › nutrients-1420839-supplementary.pdf]

Supplementary table

Scheme 1. Mean Change in Study Variables at Post-Intervention and 6- and 12-Month Follow-Up Adjusted for 'Completers'.

	Study Group				Difference in change score		
	Control		Intervention		(Group 1 - Group 0)		
**Outcome**	**n**	**M (SD)**	**n**	**M (SD)**	**M (95% CI)**	**Effect size**	**p (two-tailed)**
**Primary and secondary outcomes**							
Weight/shape concerns							
Baseline	229	1.62 (1.70)	254	1.46 (1.52)			
Post-intervention	228	1.54 (1.66)	257	1.31 (1.47)	−0.07 [−0.25, 0.11]	−0.044	0.441
6 month follow-up	227	1.69 (1.71)	256	1.60 (1.63)	0.08 [−0.13, 0.29]	0.050	0.473
12 month follow-up	228	1.67 (1.69)	256	1.76 (1.62)	0.23 [−0.01, 0.47]	0.143	0.057
State body satisfaction							
Baseline	229	64.75 (32.02)	249	68.44 (29.53)			
Post-intervention	229	66.52 (32.34)	257	69.25 (29.41)	−1.07 [−5.02, 2.87]	−0.035	0.594
6 month follow-up	226	67.03 (31.86)	257	67.29 (30.60)	−2.61 [−7.04, 1.82]	−0.085	0.249
12 month follow-up	228	64.88 (31.03)	255	67.25 (29.56)	−0.86 [−5.90, 4.18]	−0.028	0.738
Dietary restraint							
Baseline	228	1.91 (0.94)	257	1.87 (0.93)			
Post-intervention	229	1.78 (0.93)	259	1.72 (0.84)	−0.02 [−0.13, 0.08]	−0.021	0.692
6 month follow-up	228	1.92 (1.06)	257	1.84 (0.94)	−0.05 [−0.20, 0.10]	−0.053	0.518
12 month follow-up	227	1.86 (0.99)	257	1.90 (1.00)	0.07 [−0.09, 0.24]	0.075	0.397
Drive to increase muscularity							
Baseline	229	10.82 (5.07)	255	10.53 (5.22)			
Post-intervention	230	9.66 (4.75)	257	9.72 (4.87)	0.38 [−0.32, 1.08]	0.074	0.284
6 month follow-up	229	10.15 (4.98)	257	10.28 (4.72)	0.41 [−0.42, 1.25]	0.080	0.332
12 month follow-up	229	9.52 (4.75)	256	10.59 (5.06)	1.39 [0.44, 2.34]	0.270	0.004
Self-esteem							
Baseline	228	11.28 (2.95)	253	11.06 (2.61)			
Post-intervention	227	11.28 (2.97)	255	10.93 (3.02)	−0.10 [−0.53, 0.33]	−0.036	0.655
6 month follow-up	230	10.89 (3.12)	258	11.00 (3.04)	0.35 [−0.15, 0.84]	0.126	0.174
12 month follow-up	227	10.77 (3.25)	256	10.73 (3.03)	0.24 [−0.30, 0.78]	0.086	0.384
Depressive symptoms							
Baseline	226	15.83 (6.82)	256	15.16 (6.84)			
Post-intervention	230	16.13 (7.36)	257	15.49 (7.14)	0.09 [−0.90, 1.08]	0.013	0.863
6 month follow-up	230	17.79 (8.74)	258	16.10 (7.56)	−0.97 [−2.13, 0.19]	−0.142	0.102
12 month follow-up	228	17.74 (8.43)	255	17.92 (8.17)	0.89 [−0.37, 2.16]	0.130	0.167
**Exploratory outcomes**							
Thin ideal internalization							
Baseline	229	11.70 (5.09)	256	11.52 (4.87)			
Post-intervention	228	11.77 (4.94)	259	11.57 (4.66)	0.01 [−0.71, 0.73]	0.002	0.982
6 month follow-up	226	12.38 (5.26)	257	11.78 (4.83)	−0.44 [−1.25, 0.38]	−0.088	0.294
12 month follow-up	228	12.25 (5.05)	256	12.24 (4.74)	0.17 [−0.67, 1.01]	0.034	0.689
Muscular internalization							
Baseline	229	11.45 (5.47)	258	11.24 (5.30)			
Post-intervention	230	11.21 (5.68)	259	10.57 (5.28)	−0.46 [−1.24, 0.33]	−0.085	0.255
6 month follow-up	229	11.49 (5.71)	257	11.51 (5.51)	0.21 [−0.69, 1.10]	0.039	0.653
12 month follow-up	229	11.15 (5.58)	257	12.06 (5.66)	1.10 [0.11, 2.10]	0.204	0.03
Upwards appearance comparison							
Baseline	228	11.74 (6.60)	256	10.83 (6.05)			

Post-intervention	228	11.22 (6.58)	258	10.57 (5.28)	0.39 [−0.42, 1.19]	0.062	0.344
6 month follow-up	229	12.24 (6.98)	257	11.53 (6.33)	0.21 [−0.78, 1.19]	0.033	0.679
12 month follow-up	228	12.43 (6.83)	257	12.27 (6.57)	0.73 [−0.28, 1.74]	0.116	0.154

Effects are based on multiple imputation (n = 50 imputations). Differences compare post-intervention to all other timepoints, adjusted for age, language, and school type.
